# Increased Coupling of Intrinsic Networks in Remitted Depressed Youth Predicts Rumination and Cognitive Control

**DOI:** 10.1371/journal.pone.0104366

**Published:** 2014-08-27

**Authors:** Rachel H. Jacobs, Lisanne M. Jenkins, Laura B. Gabriel, Alyssa Barba, Kelly A. Ryan, Sara L. Weisenbach, Alvaro Verges, Amanda M. Baker, Amy T. Peters, Natania A. Crane, Ian H. Gotlib, Jon-Kar Zubieta, K. Luan Phan, Scott A. Langenecker, Robert C. Welsh

**Affiliations:** 1 Department of Psychiatry, University of Illinois at Chicago, Chicago, Illinois, United States of America; 2 Department of Psychiatry, University of Michigan, Ann Arbor, Michigan, United States of America; 3 Research and Development Program, Jesse Brown VA Medical Center, Chicago, Illinois, United States of America; 4 VA Ann Arbor Healthcare System, Ann Arbor, Michigan, United States of America; 5 Department of Psychology, Stanford University, Stanford, California, United States of America; 6 Department of Radiology, Molecular and Behavioral Neuroscience Institute, Ann Arbor, Michigan, United States of America; 7 Departments of Psychology and Anatomy & Cell Biology, University of Illinois at Chicago, Chicago, Illinois, United States of America; 8 Mental Health Service Line, Jesse Brown VA Medical Center, Chicago, Illinois, United States of America; Max Planck Institute for Human Cognitive and Brain Sciences, Germany

## Abstract

**Objective:**

Functional connectivity MRI (fcMRI) studies of individuals currently diagnosed with major depressive disorder (MDD) document hyperconnectivities within the default mode network (DMN) and between the DMN and salience networks (SN) with regions of the cognitive control network (CCN). Studies of individuals in the remitted state are needed to address whether effects derive from trait, and not state or chronic burden features of MDD.

**Method:**

fcMRI data from two 3.0 Tesla GE scanners were collected from 30 unmedicated (47% medication naïve) youth (aged 18–23, modal depressive episodes = 1, mean age of onset = 16.2, SD = 2.6) with remitted MDD (rMDD; modal years well = 4) and compared with data from 23 healthy controls (HCs) using four bilateral seeds in the DMN and SN (posterior cingulate cortex (PCC), subgenual anterior cingulate (sgACC), and amygdala), followed by voxel-based comparisons of the whole brain.

**Results:**

Compared to HCs, rMDD youth exhibited hyperconnectivities from both PCC and sgACC seeds with lateral, parietal, and frontal regions of the CCN, extending to the dorsal medial wall. A factor analysis reduced extracted data and a PCC factor was inversely correlated with rumination among rMDD youth. Two factors from the sgACC hyperconnectivity clusters were related to performance in cognitive control on a Go/NoGo task, one positively and one inversely.

**Conclusions:**

Findings document hyperconnectivities of the DMN and SN with the CCN (BA 8/10), which were related to rumination and sustained attention. Given these cognitive markers are known predictors of response and relapse, hyperconnectivities may increase relapse risk or represent compensatory mechanisms.

## Introduction

Studying individuals with a history of major depressive disorder (MDD) who are currently in the remitted state allows for a unique examination of potential trait-based mechanisms of depression and depression relapse (e.g., [1]). As such, phenotypic expressions assessed during remission may represent reliable markers of illness course, offering refined targets for future research among high-risk cohorts. Studying putative mechanisms early in the course of MDD (avoiding the chronic burden of repetitive illness scarring), during the remitted state (avoiding state effects), and towards the end of development (avoiding developmental variability in early adolescence) can provide a clearer understanding of mechanisms in relapse and recurrence given risk for depressive relapse increases as a function of previous episodes [2] and may result in greater neurobiological insults (e.g., [3]). Importantly, mechanisms identified through this approach can inform the development of early detection and primary and secondary prevention programs.

One method for understanding trait-based markers for MDD involves studying network function through measurements of network connectivity. Resting state fMRI has emerged as an approach for the identification of brain-based biomarkers, particularly in the detection of variations in network connectivity deriving from clinical features [4]. Moreover, resting state fMRI has emerged as a useful technique for studying psychiatric populations due to good signal to noise ratios, reduced participant burden, and lends itself to clinical translation [5]. Disrupted network connectivity has been documented among individuals within a major depressive episode (MDE [6], [7]). In particular, disturbances in a set of regions including the posterior cingulate cortex (PCC), medial prefrontal cortex (mPFC), and inferior parietal cortex (IPC) have been reported and are hypothesized to contribute to depression [8], [9]. These regions are included in a task negative default mode network (DMN), which encompasses regions demonstrating decreases in activation during performance of attention-demanding tasks and corresponding increases in activation during rest, mind-wandering, or during self-reflective thought (for a review see [10]).

In contrast, a task positive network includes regions that increase in activation during attention to demanding tasks [11]. Task positive and task negative networks act in opposition, as they have been shown to be anticorrelated during both cognitive tasks and during the resting state. Two dissociable task positive networks include the Cognitive Control Network (CCN) and salience network (SN; [12]). The SN supports emotion processing and autonomic regulation and incorporates regions such as the dorsal anterior cingulate cortex (dACC) and the orbital frontoinsula [12].

Resting state fMRI examines intrinsic network functioning by capturing temporal correlations between brain regions in the blood oxygen level-dependent (BOLD) signal, offering a reliable [13]–[15] method to link neural networks to traits that may make an individual vulnerable to relapse. These spontaneous low-frequency (<.1 Hz) fluctuations yield maps of neural systems that make up an individual's functional connectome [16]. The task positive network (SN, CCN) can also be studied during the resting state. Regions of the SN, including the subgenual anterior cingulate cortex (sgACC), have been observed to be hyperconnected at rest among individuals with MDD [7] and low frequency oscillations in resting state networks support biases in information processing [17]. As these networks interact and may even compete with one another to modulate attention to both the external and internal worlds [18], and given that the SN in particular may initiate switching between the CCN and DMN; understanding network functioning during rest will advance the study of MDD. For example, observed deficits in task-related activation among individuals with psychiatric disorders has led to the hypothesis that aberrant connectivity is a core feature of mental illness [19], but the lack of consistency across task-based fMRI experiments has slowed progress towards integrated models of network dysfunction in psychopathology. Aberrant network functioning is likely to underlie and support observable clinical symptoms such as rumination (DMN) and emotional reactivity (SN e.g., [8]) consistent with network models of psychopathology [19].

Increased connectivity within and between networks is hypothesized to contribute to the tendency of depressed individuals to attend to internal stimuli and to return inadvertently to internal thoughts at the expense of external tasks [6], [20],[21]. Moreover, depressive symptoms including maladaptive levels of internally focused thought have been associated with increased DMN connectivity [22],whereas dysfunction in the SN contributes to biases in emotion processing and autonomic regulation ([23], [24], for a review see [25]). Unmedicated adults with MDD demonstrate increased connectivity of the DMN with the SN [6]. In addition, the dorsal medial and dorsal lateral portion of the PFC may represent a nexus in dysfunctional depression-associated connectivity between all three networks [22]. To date; however, the link between connectivity-related abnormalities and clinical and course features remains relatively unexplored with few positive results (but see [21]). Furthermore, only limited work has explored whether these differences exist during periods of remission [26]–[28]. No studies to date have been conducted with remitted late-adolescent or early adult samples – this developmental epoch may serve as a critical assessment and intervention period of near-complete neural development and network maturation prior to chronic disease progression and sequelae. Finally, investigators have explored preliminary links between network dysfunction, rumination, and cognitive control [29]; however, no studies to date directly link these mechanisms.

Thus, a critical unanswered question is whether abnormal neural connectivities represent trait markers of vulnerability to illness and recurrence that are not related to state or chronic illness scars. To address this issue, we examined functional connectivity among youth in a remitted state. This design mitigates several potential confounds including current mood state, illness severity, increased scar due to repeated episodes, and the effects of current medication. We hypothesized that hyperconnectivities would be observed within and between regions of the DMN and SN with regions of the CCN among unmedicated youth with rMDD compared to Healthy Controls (HCs), reflecting potential trait-based risk for relapse, or conversely resilience. Finally, given preliminary research correlating DMN functional connectivity with repetitive negative thoughts such as rumination (e.g., [21]) as well as evidence that the sgACC contributes to ruminative processes [30], we conducted exploratory analyses testing whether self-report rumination was correlated with aberrant connectivities among rMDD youth. Last, in order to specifically probe the clinical and behavioral correlates of hyperconnectivity with the CCN, we conducted exploratory correlations of connectivity measures with performance on the Parametric Go/No-Go Task [31], [32] in light of previous work suggesting inhibitory control predicts course of illness among adults with MDD [33].

## Methods

### Participants

The current study was approved by the University of Michigan (UM) and the University of Illinois at Chicago (UIC) Institutional Review Boards and all participants signed consent. Participants were recruited using flyers and multiple forms of posting on the internet. All participants completed an identical assessment protocol, including the Diagnostic Interview for Genetic Studies (DIGS; [34]), the Hamilton Depression Scale (Ham-D; [35]), the Parametric Go/No-Go Test [31], [32], and the Ruminative Responsiveness Scale (RRS, [36]). Participants were considered remitted from MDD if they previously met criteria for at least one MDE, but currently scored below a 7 on the Ham-D (administered during the phone screen and during the initial diagnostic interview). HCs could not meet current or past criteria (Never Mentally Ill, NMI) for MDD or any other Axis I or II psychiatric disorder and had no first degree relatives with a history of psychiatric illness. In addition, participants were required to be medication free for a period of 30 days prior to the scan and those with substance abuse or dependence within the past six months were excluded. Diagnosis of past MDD or NMI was determined with DIGS, and confirmed using a modified Family Interview for Genetic Studies completed with a parent or guardian [34]. The final sample included 30 rMDD (19 UM, 11 UIC) and 23 healthy controls (HCs; 16 UM, 7 UIC) between the ages of 18–23 years (66% Female). Participant demographics and clinical characteristics are presented in Table 1. Previous treatment history within the rMDD group (data available for n = 19) included medication (n = 13) and psychotherapy (n = 17).

**Table 1:** Sample Demographics and Clinical Characteristics.

	HC (n = 23)	rMDD (n = 30)
	M (SD)	M (SD)
**Age**	20.8 (1.6)	21.0 (1.5)
**Education**	14.9 (1.2)	14.5 (1.4)
HamD*	0.5 (1.1)	4.1 (7.2)
**VIQ**	109.0 (8.5)	109.7 (9.2)
RRS Total*	29.8 (8.5)	44.4 (15.7)
RRS Brooding*	6.8 (2.1)	9.0 (4.0)
RRS Depression Related*	16.1 (4.7)	25.1 (9.0)
RRS Reflection*	6.9 (2.7)	10.3 (3.9)
**Modal Years Well**	n/a	4
**Modal MDE Episodes**	n/a	1
**% Medication Naïve**	n/a	47%
**Age of First Onset**	n/a	16.2(2.6)
**Age of Most Recent Episode**	n/a	18.5(2.2)
**Longest MDE Duration (weeks)**	n/a	31.8(38.4)
**Comorbid Anxiety Diagnosis** ∧	2	8
**Modal Number of Psychiatric Hospitalizations**	n/a	0

*Note.* HC = Healthy Control, rMDD = remitted Major Depressive Disorder; M = Mean; SD = Standard Deviation; HamD = Hamilton Depression Rating Scale; VIQ = Verbal IQ; RRS = Ruminative Response Scale; MDE = Major Depressive Episode.

∧ Specific Phobia was not an exclusion for NMI.

### Rumination

Self-report rumination was collected using the Ruminative Response Scale (RRS; [36]). Forty individuals (23 rMDD, 17 HC) completed the RRS. Individuals with scores greater than two standard deviations from the mean were given a truncated score [37]. Correlations were computed between total rumination score and extracted ROI values for regions that differed significantly between groups in seed-based connectivity.

### Parametric Go/No-Go

This task (described previously, [31]) was administered to all subjects during cognitive testing prior to the scan to assess sustained attention accuracy (Go Accuracy), context-based inhibition (No-Go Accuracy), and processing speed (Reaction Time, [32]), all aspects of cognitive control. Two outliers (1 HC, 1 rMDD) were given a truncated performance score for Go Accuracy (two SDs, [37]). Exploratory correlations were computed using factors from extracted ROIs with these measures of cognitive control.

### fcMRI Acquisition

At UM an eyes-open resting state scan was acquired over eight minutes on a 3.0 T GE Signa scanner (Milwaukee, WI) using T2*-weighted single shot reverse spiral sequence with the following parameters: 90 degree flip, field-of-view 20, matrix size = 64×64, slice thickness = 4 mm, 30 ms echo time, 29 slices. Eyes-open, resting scans at UIC were collected over eight minutes on a 3.0 T GE Discovery scanner (Milwaukee, WI) using parallel imaging with ASSET and T2* gradient-echo axial EPI with the following parameters: 90 degree flip, field-of-view 22, matrix size = 64×64, slice thickness = 3 mm, 22.2 ms echo time, 44 slices. At both sites, high-resolution anatomic T1 scans were obtained for spatial normalization and motion was minimized with foam pads, a visual tracking line (UIC only) and/or cross (UIC and UM) on the display, and by conveying the importance of staying still to participants, with TRs of 2000 ms and 240 TRs total. Site effects of acquisition parameters and scanner were evaluated and are reported in Supplementary Material (Figure S2).

### fc-MRI Preprocessing

Several steps were taken to reduce potential sources of noise and artifact. Slice timing was completed with SPM8 (http://www.fil.ion.ucl.ac.uk/spm/doc/) and motion detection algorithms were applied using FSL (http://fsl.fmrib.ox.ac.uk/fsl/fslwiki/). Coregistration of structural images to functional images was followed by spatial normalization of the coregistered T1-spgr to the Montreal Neurological Institute (MNI) template. The resulting normalization matrix was then applied to the slice-time-corrected, physiologically corrected, time series data. These normalized T2* time-series data were spatially smoothed with a 5 mm Gaussian kernel resulting in T2* images with isotropic voxels, 2 mm on a side.

### Cross-Correlation Analysis

Time series were detrended and mean centered. Physiologic correction was performed by regressing out white matter and cerebral spinal fluid signals [38]. Motion parameters were regressed out [39]. Based upon the recent literature [39], [40], motion volumes were identified based on any TR to TR movement exceeding .5 mm and did not differ between groups (Figure S1) [40]. All significant differences were evaluated with respect to movement, and movement did not influence significant differences identified using the PCC and sgACC seeds. However, connectivities with the left amygdala seed became non-significant after covarying movement measures. Global signal was not regressed due to colinearity violations with gray matter signal, problematic mis-estimates of anticorrelations [41], and because it does not affect distance-micromovement relationships [39]. Finally time series were band-pass filtered over 0.01–0.10 Hz. Seeds were derived based on previous literature examining resting state connectivity of the amygdala [42], [43], PCC [44], [45], and sgACC [46], [47]. The following coordinates were used: PCC (DMN, −5/5, −50, 36), amygdala (SN, −23/23, −5, −19), sgACC (SN, −4/4, 21, −8). Regions of Interest (ROIs; 2.9 mm radius, 19 voxels) were defined in MNI space and spatially averaged time course data were extracted from ROIs for each participant. Seeds were overlaid on the average warped structural anatomy of the current sample and adjusted where necessary.

Correlation coefficients were calculated between mean time course for seed regions and all other voxels of the brain, resulting in a 3-dimensional correlation coefficient image (r image). These r images were transformed to z scores using a Fisher transformation. Resulting z images were used in 2-sample Student *t* tests implemented in SPM8. AlphaSim was used with 1000 Monte Carlo simulations to determine whole brain correction with a joint threshold of height and extent (*p*<.005, cluster extent of 440 mm^3^) for group comparisons with a corrected *p* value of .05. Images are displayed on an averaged brain anatomy derived from the current sample.

Importantly, movement was addressed using regression of white matter signal as recommended in the recent literature [39], [40]. Further, we conducted additional analyses (see Table S3 in File S1 and Figure S1) to fully explore potential (micro) movement confounds. All PCC and sgACC clusters reported remained significant when subjects with any TR to TR movements greater than .5 mm (typically Z displacement) were excluded.

### Exploratory Factor Analysis

Factor analysis is a statistical technique that can be used to discover which variables form coherent subsets that are somewhat independent. Factors are hypothesized to reflect underlying processes resulting in correlations between variables. Factor analysis differs from principal components analysis in that factor analysis examines shared variance only and attempts are made to estimate and eliminate variance that derives from error [37]. Factor analysis can be effective in data reduction and may also segregate noise from connectivity signal in fMRI [23]; therefore, exploratory factor analysis was conducted on extracted z values from each cluster of significant differences between groups separately for each seed region. The number of factors retained was determined using maximum likelihood as an extraction method and a threshold of eigenvalue >1, followed by oblique rotation. We also verified that the extracted factors surpassed 50% of total variance.

## Results

### PCC connectivity

**Figure 1:**
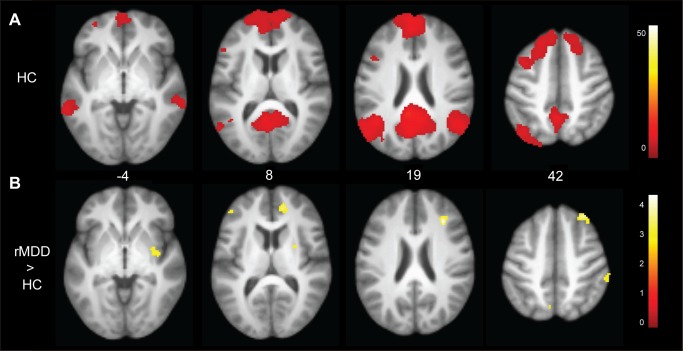
Connectivity of left posterior cingulate seed and between group differences. Panel A: Connectivities among HC youth illustrate the default mode network. Panel B: Youth with remitted depression demonstrated greater connectivity with the right insula, superior and middle frontal gyrus, putamen, angular gyrus, and left middle frontal gyrus.

Across groups, the left PCC seed was correlated with regions encompassing the DMN including the medial prefrontal cortex (PFC), posterior and superior temporal gyri, and bilateral hippocampus. Figure 1 illustrates the DMN among HC participants as well as connectivities that were greater among rMDD compared to HC participants. Table 2 details significant differences between groups. Compared to HCs, youth with rMDD demonstrated greater connectivity from the left PCC seed to the right insula and right superior and middle frontal gyrus (BA 9), as well as the left precuneus (BA 7) and left dorsolateral PFC (BA 8/10). Results for the right PCC are included as Supplementary Material for comparison (Table S1 in File S1).

**Table 2:** Differences between Healthy Controls and remitted Major Depression for left posterior cingulate seed.

Lobe/region	BA	x	y	z	Z	mm^3^	Factor
**Frontal**							
**Superior/Middle frontal**	10	12	45	14	3.24	832	PCC 2*
	9	27	24	20	3.85	824	PCC1
	8	26	32	42	3.63	728	PCC 1, 2
**Inferior frontal**	46	−41	35	12	3.53	688	PCC 1
**Parietal**							
**Precuneus**	7	−11	−63	30	3.82	1376	PCC 1
	7	−6	−63	49	3.41	712	PCC 1
**Inferior Parietal**	40	43	−36	30	3.9	864	PCC 1
**Subcortical**							
**Lentiform nucleus**		17	−9	3	3.57	1384	PCC 1

Note. BA = Brodmann's Area; mm = millimeter; PCC = posterior cingulate cortex;

*PCC Factor 2 was significantly inversely correlated with Rumination Total Score, r = −.49, *p* = .03.

x, y, and z coordinates are converted and reported in Talairach space.

### sgACC connectivity

**Figure 2:**
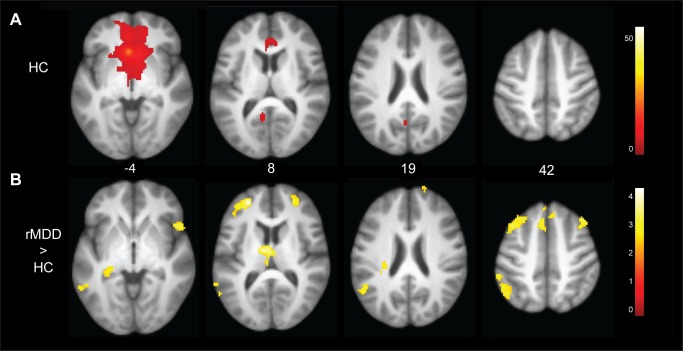
Connectivity of the left subgenual anterior cingulate seed and between group differences. Panel A: Connectivities among HC youth illustrate the salience network. Panel B: Youth with remitted depression demonstrated greater connectivity from the left subgenual anterior cingulate seed to the right anterior inferior frontal gyrus/insula, bilateral medial, superior and middle frontal cortex, thalamus, and left medial temporal gyrus, inferior parietal lobule, and parahippocampal gyrus.

Across groups, left sgACC activation was significantly correlated with nearby areas including the orbital frontal cortex, thalamus, hippocampus, and the PCC. Figure 2 illustrates the SN among HC participants and connectivities that were greater among rMDD youth compared to HCs. Table 3 details these significant differences. rMDD youth demonstrated greater connectivity from the left sgACC seed to the bilateral superior and medial frontal cortex (BA 8/10), cerebellum, and thalamus, as well as the left medial temporal gyrus (BA 39/40), parahippocampus, and the right operculum/anterior insula when compared to HCs. Results for the right sgACC are included as Supplementary Material for comparison (Table S2 in File S1).

**Table 3:** Differences between Healthy Controls and remitted Major Depression for left subgenual anterior cingulate seed.

Lobe/region	BA	x	y	z	Z	mm^3^	Factor
**Frontal**							
**Superior/Middle frontal**	10	10	63	19	3.29	1400	sgACC 1
	8	36	24	41	3.52	2832	sgACC 1
	8	8	32	49	3.72	3664	sgACC 1,2*
	8	−36	16	45	3.58	2240	sgACC 2*
	9	−24	43	13	4.19	1944	sgACC 1,2*
	8	4	45	39	3.54	680	
**Inferior frontal**	47	43	14	−3	3.36	856	sgACC 2*
**Limbic**							
**Parahippocampus**	30	−22	−38	0	3.67	728	sgACC 3*
**Temporal**							
**Inferior Temporal**	37	−54	−54	−1	3.71	1536	sgACC 2*
	21	−57	−15	−19	3.51	448	sgACC 2*
**Parietal**							
**Postcentral**	3	31	−36	49	3.12	544	sgACC 3*
**Angular**	39	−43	−65	35	3.81	8392	sgACC 1
**Subcortical**							
**Medial dorsal nucleus**		−1	−15	6	4.01	1760	sgACC 3*
**Caudate tail**		−24	−30	16	3.42	592	sgACC 3*
**Cerebellum**							
**Pyramis**		−31	−83	−33	3.89	3936	sgACC 1
**Inferior semi-lunar lobule**		29	−71	−39	3.56	1088	sgACC 1
		15	−69	−39	3.3	1328	sgACC 1

*Note*. BA = Brodmann's Area; mm = millimeter; sgACC = subgenual anterior cingulate cortex;

*sgACC Factor 2 was positively correlated with Go Accuracy (r = .40, *p* = .04) and sgACC Factor 3 was negatively correlated with No-Go Accuracy (r = −.44, *p* = .02).

x, y, and z coordinates are converted and reported in Talairach space.

### Amygdala hyperconnectivity

**Figure 3:**
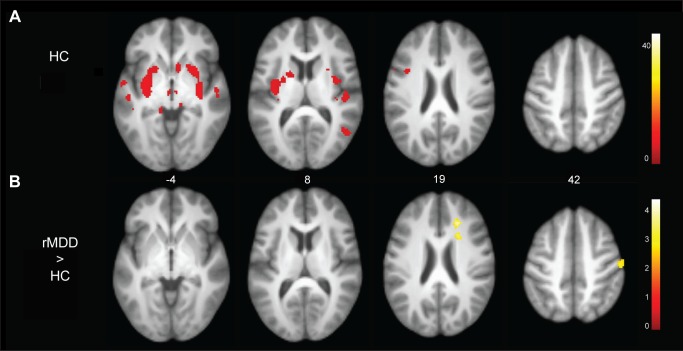
Connectivity of the left amygdala seed and between group differences. Figure 3 Panel A: Connectivities among HC youth with the left amygdala seed. Panel B: Youth with remitted depression demonstrated hyperconnectivities with the right medial frontal gyrus, medial parietal lobe, postcentral gyrus, and anterior cingulate cortex.

Across groups, left amygdala activation was significantly correlated with surrounding bilateral amygdaloid, hippocampal, and uncal regions. Figure 3 illustrates regions of the SN significantly connected with the left amygdala seed among HCs and those that were significantly different between rMDD and HC participants. Table 4 details these differences. The rMDD group exhibited greater connectivity between the left amygdala and the right medial frontal gyrus, medial parietal lobe, rostral ACC, and left parahippocampal gyrus (all regions not displayed in Figure 3). There were no significant between group differences for connectivity with the right amygdala.

**Table 4:** Differences between Healthy Controls and remitted Major Depression for left amygdala seed.

Lobe/region	BA	x	y	z	Z	mm^3^
**Parietal**						
**Postcentral**	3	48	−19	39	3.04	648
**Limbic**						
**Anterior Cingulate**	32	19	24	19	4.22	784
**Uncus**	36	−18	−9	−27	3.81	656
**Subcortical**						
**Caudate body**		19	12	21	3.82	520

*Note.* BA = Brodmann's Area; mm = millimeter; amygdala clusters did not converge in factor analysis. x, y, and z coordinates are converted and reported in Talairach space.

### Data Reduction with Exploratory Factor Analysis

Results from exploratory factor analysis suggested a two factor solution for the PCC seed and a three factor solution for the sgACC seed. PCC Factor 1 had higher loadings on six of eight clusters, as indicated in Table 2. PCC Factor 2 had a higher loading with the right middle and medial frontal gyri and comparable loadings on a second right middle frontal gyrus cluster. sgACC Factor 1 included 11 of the 17 clusters that were hyperconnected among rMDD youth, as indicated in Table 3. Four clusters had dominant loadings on sgACC Factor 2, and four clusters had dominant loadings on sgACC Factor 3. As detailed in Tables 2 and 3, some clusters loaded highly on more than one factor. Factor analysis of the amygdala-based connectivity clusters failed to converge.

### Relation of Intrinsic Networks to Rumination and Cognitive Control

**Figure 4:**
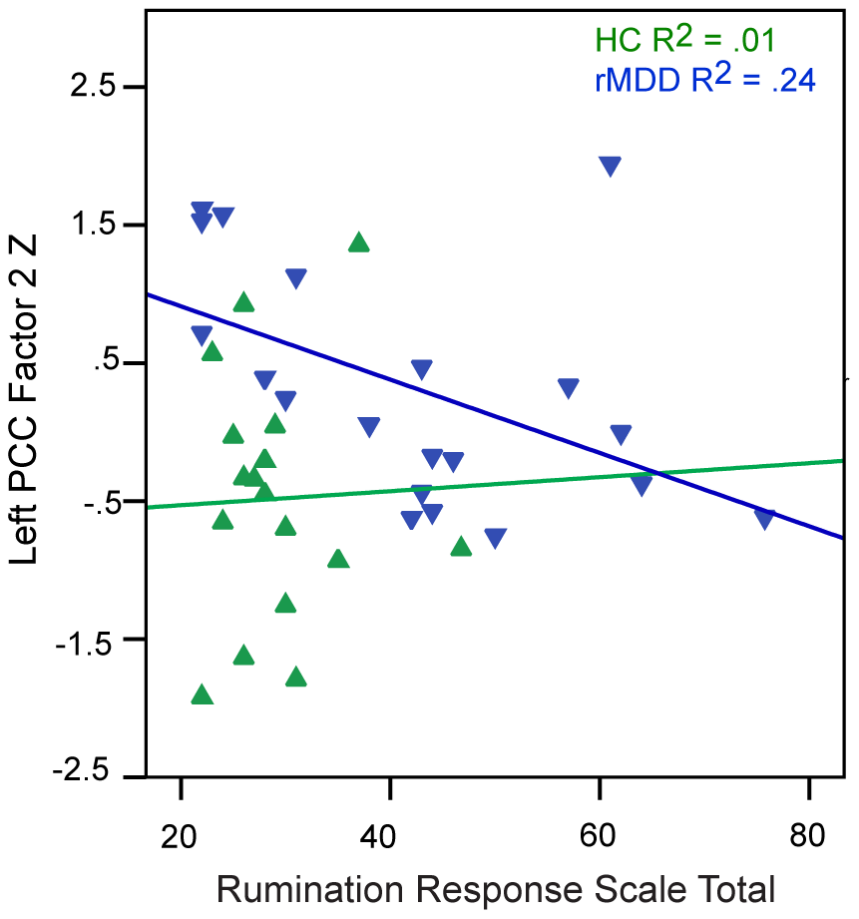
Correlation of Seed-based Factor with Rumination. Correlation of the left posterior cingulate Factor 2 with Rumination.

**Figure 5:**
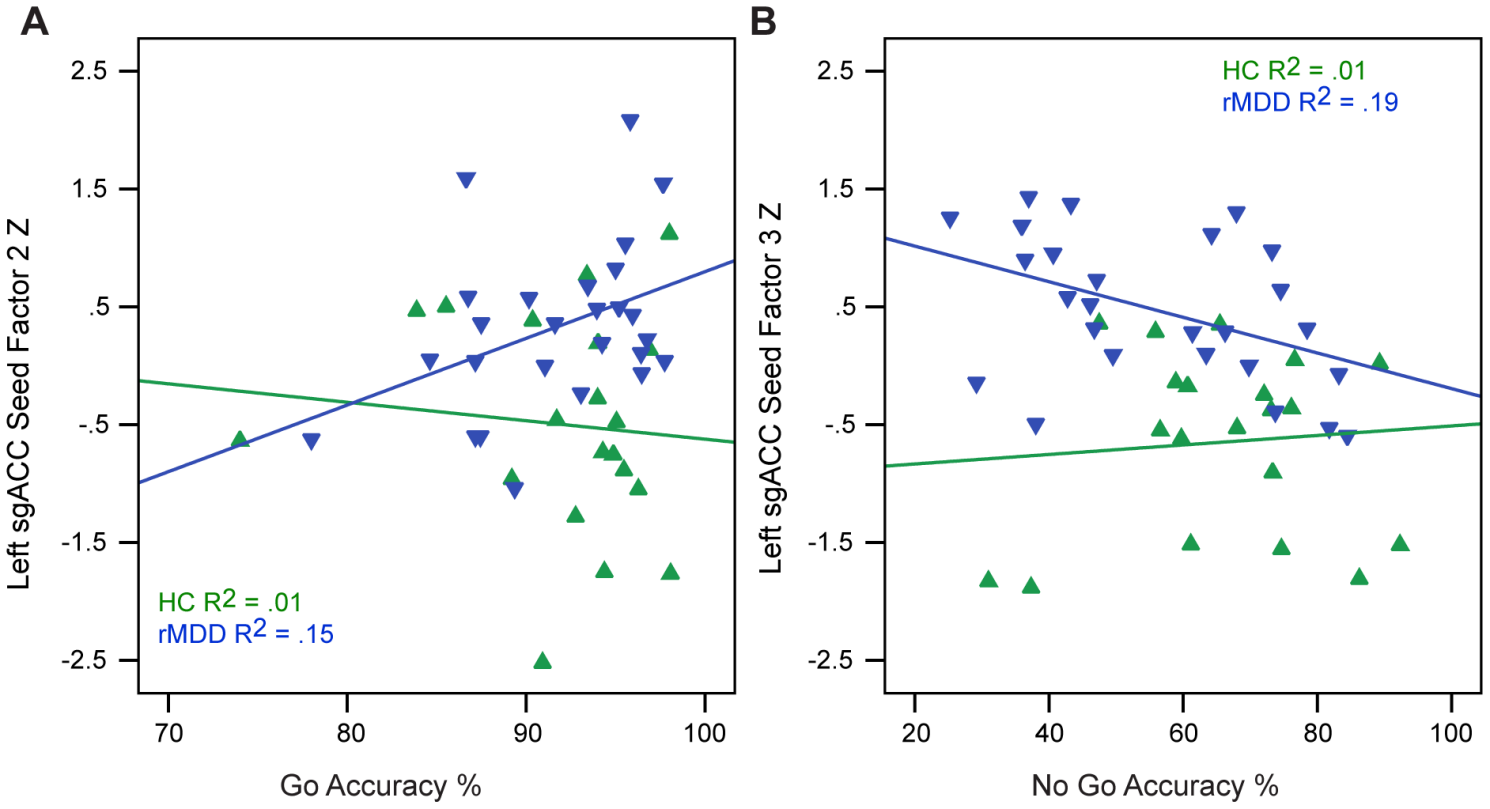
Correlation of Seed-based Factor with Rumination and Cognitive Control. A) Correlation of the left subgenual anterior cingulate Factor 2 with Go Accuracy and B) correlation of left subgenual anterior cingulate Factor 3 with No-Go percent accuracy.

As expected, rMDD youth reported higher levels of rumination than HCs (t = −3.78, df = 38, *p*<.01). For the parametric Go/No-Go, rMDD youth exhibited greater impulse control problems (No-Go Accuracy, t = 2.30, df = 49, *p* = .03), but did not differ in Go Accuracy (t = −0.59, df = 49, *p* = .99) or Go Response Time (t = −0.70, df = 49, *p* = .47). Among youth with rMDD, Go Response Time was inversely correlated with rumination (r = −.48, *p*<.05). Also among rMDD youth, the PCC Factor 2 (right superior and middle frontal gyrus connectivity) was inversely correlated with rumination (r = −.49, *p* = .03; Figure 4). Among rMDD youth only, the sgACC Factor 2 (left middle frontal, inferior temporal, right inferior frontal, and middle frontal gyri connectivity) was positively associated with Go Accuracy (r = .40, *p* = .04; Figure 5, Panel 1) and sgACC Factor 3 (left parahippocampal gyrus, caudate, bilateral dorsal medial thalamus, right postcentral gyri connectivity) was inversely correlated with No-Go Accuracy (r = −.44, *p* = .02; Figure 5, Panel B).

### Potential Confounds

To rule out potential confounds, we conducted post-hoc exploratory analyses of clinical features and technological challenges and also examined the influence of participant sex (see Supplementary Material). These factors did not significantly affect between-group results for the PCC and sgACC seeds.

## Discussion

The current results address an understudied, yet very important set of questions regarding the dissociation of state, trait, compensatory, and scar features of MDD. To begin unravelling these questions, we obtained resting state data from a homogeneous sample of carefully characterized, unmedicated youth in a remitted state, allowing us to study MDD course in the absence of state and chronic burden features that may have confounded previous results. This is the first study of young adults with rMDD and we demonstrate hyperconnectivity of both the DMN and SN with portions of the right superior and middle frontal gyri – a central node in the CCN. Furthermore, observed hyperconnectivities were related to two known predictors of relapse: rumination (inverse correlation) and sustained attention (positive correlation).

Self-report rumination has previously been documented to correlate with sgACC-PCC connectivity during task among actively depressed adults [29]. We found an inverse correlation of rumination with hyperconnectivities suggesting that these increased temporal correlations may be protective, compensatory, or may even reduce risk for relapse. In contrast, another hypothesis is that over-enriched connections to regions outside of the CCN may be detrimental. For example, hyperconnectivity to a factor from the left sgACC to regions including the left parahippocampal gyrus, caudate, bilateral dorsal medial thalamus, and right postcentral gyri was associated with poorer inhibitory control. When interpreted in light of the strengths of the current sample, our results offer new evidence that connectivity differences may represent both protective and trait-based risk factors for relapse in MDD course.

The sgACC has long been implicated as a dysfunctional node in acute MDD and has been correlated with negative mood states, treatment response, and “treatment refractoriness” [48]–[50]. Our finding that this SN node is hyperconnected with both the DMN and EN among rMDD youth is noteworthy. Aberrant sgACC functioning and connectivity have been conceptualized as a *consequence* of depressive illness and sadness, as opposed to a potential risk or protective factor for future MDEs. In fact, a recent investigation of actively depressed, unmedicated adolescents found that higher levels of rumination were associated with *reduced* connectivity between the sgACC and the medial frontal gyrus [7]. Our findings suggest that *increased* connectivity between these same regions may strengthen the adolescent's ability to resist rumination and support attentional control, thereby promoting and potentially sustaining remission. Remitted youth whose CCN is overly-engaged may more effectively downregulate mind-wandering, self-referential thought supported by the DMN.

Our results extend and contextualize findings of a proposed abnormal ‘dorsal nexus’ in MDD [22] by suggesting that hyperconnectivity of the DMN and SN with the CCN is an early course marker observable outside of MDEs, similar to one study of DMN function among remitted preschool children [28]. The superior and middle frontal gyri may represent an extension of the CCN that modulates the DMN and SN among those with a history of MDD, which may function to ameliorate trait-based ruminative tendencies that often remain into periods of wellness [51], [52].

It is also worth noting, however, that the primary factors (for both the PCC and sgACC seeds) accounting for the majority of factor variance across networks were not related to clinical features of illness or functioning in the current study – our results may reflect trait or scar biomarkers. A trait biomarker would represent a characteristic observable prior to illness and initially independent of illness, whereas a scar biomarker is a result of illness and represents a failure to achieve complete inter-episode recovery. The second and third factors, accounting for less variance, and more likely to be sample-specific, were related to cognitive markers of illness. Hyperconnectivities of the DMN and SN with portions of the CCN may result in increased neural resources directed towards emotion regulation among vulnerable individuals. Developmental hyperconnectivities may be protective to an extent, but may not always be sufficient for mounting adaptive responses to the increased adversity and stressors experienced by this population at-risk for multiple MDEs and comorbidities. We are currently following this sample longitudinally and will be able to identify predictors of both relapse and resilience.

We note several limitations of our study. First, the current data cannot discriminate between network abnormalities that render an individual vulnerable to the first-onset of MDD versus scar consequences of illness, normal maturation processes, or even protective compensatory mechanisms. In this context, prospective studies of high-risk cohorts represent an important direction for future research. Second, despite the strength of recruiting a medication-free sample, we could not examine how previous medication use or therapy exposure may have affected current results. As a related point, because our participants have had relatively fewer MDEs and have been well for relatively extended periods of time, they may represent a more mild severity or course of illness. To capture developmental trajectories that contribute to resiliency and risk in MDD, future longitudinal research can examine whether excessive coupling of intrinsic networks predicts first-onset of depression or relapse as adolescents transition into early adulthood. In addition, anatomical specificity is of concern when conducting seed-based analyses, particularly in regions such as the PCC [53], [54]. We also did not specifically examine connectivity of the CCN using a CCN seed. We are currently analyzing the CCN during rest and task to more fully examine CCN function among remitted individuals. Future research involving larger samples will also specifically examine laterality. Last, examining network function both at rest and in response to task provides complementary and additive information regarding how networks function to support wellness or disease. Future directions could include inducing rumination during an fMRI task and examining how rumination impedes performance on cognitive control tasks to examine how these networks function together both at rest and in response to tasks relevant to the etiology of depression. Despite these limitations, we believe the examination of mechanisms during a relatively early phase of the disorder provides a level of protection against potential confounds including complex treatment histories and neural scarring resulting from decades of illness, making the current study innovative and important.

In sum, this study provides evidence of brain-based traits associated with MDD course that can be observed outside of a MDE, early in the course of illness. We believe the late adolescent-early adulthood transition represents a unique window for observing mechanisms of MDD, as stability of networks has been established developmentally, but opportunities remain for secondary prevention prior to the initiation of chronic illness. Thus, understanding the continuum of functional to dysfunctional connectivity patterns in the human brain is critical in elucidating the developmental psychopathology of MDD. Increased coupling between networks in our early course, remitted sample suggests that hyperconnectivities can be evaluated as developmental trait or resiliency factors, as targets for treatment, or as potential outcomes of early illness.

## Supporting Information

Figure S1 **Participants with movement are easily identifiable by movement deviation.** Mean movement deviations among the Healthy Control and remitted Major Depressive group in the x, y, and z planes.
(TIF)

Figure S2 **Extracted connectivity bar graphs by cluster, site, and group.** rMDD = remitted Major Depressive Disorder; HC = Healthy Control; UM = University of Michigan; UIC = University of Illinois by Chicago. Tailarach coordinates corresponding to each numbered cluster: Clusters 1–7 are connectivities with the left PCC seed, Clusters 8–24 are connectivities with the left sgACC seed, Clusters 25–28 are connectivities with the left amygdala: 1 = −41, 35, 12; 2 = −6, −63, 49; 3 = 12, 45, 14; 4 = 17, −9, 3; 5 = 26, 32, 42; 6 = 27, 24, 20; 7 = 43, −36, 30; 8 = −22, −38, 0; 9 = −24, −30, 16; 9 = −24, 43, 13; 10 = −24, 43, 13; 11 = −31, −83, −33; 12 = −36, 16, 45; 13 = −43, −65, 35; 14 = −54, −54, −1; 15 = −57, −15, −19; 16 = −1, −15, 6; 17 = 8, 32, 49; 18 = 10, 63, 19; 19 = 15, −69, −39; 20 = 29, −71, −39; 21 = 31, −36, 49; 22 = 36, 24, 41; 23 = 43, 14, −3; 24 = 4, 45, 39; 25 = −18, −9, −27; 26 = 19, 12, 21; 27 = 19, 24, 19; 28 = 48, −19, 39.
(TIF)

File S1 **Supplementary material containing supporting tables.**
(DOCX)
