# Supplementary figures and images for: Increased Coupling of Intrinsic Networks in Remitted Depressed Youth Predicts Rumination and Cognitive Control
Source: PLoS One. 2014 Aug 27;9(8):e104366. doi: 10.1371/journal.pone.0104366 (PMC4146466; doi:10.1371/journal.pone.0104366)

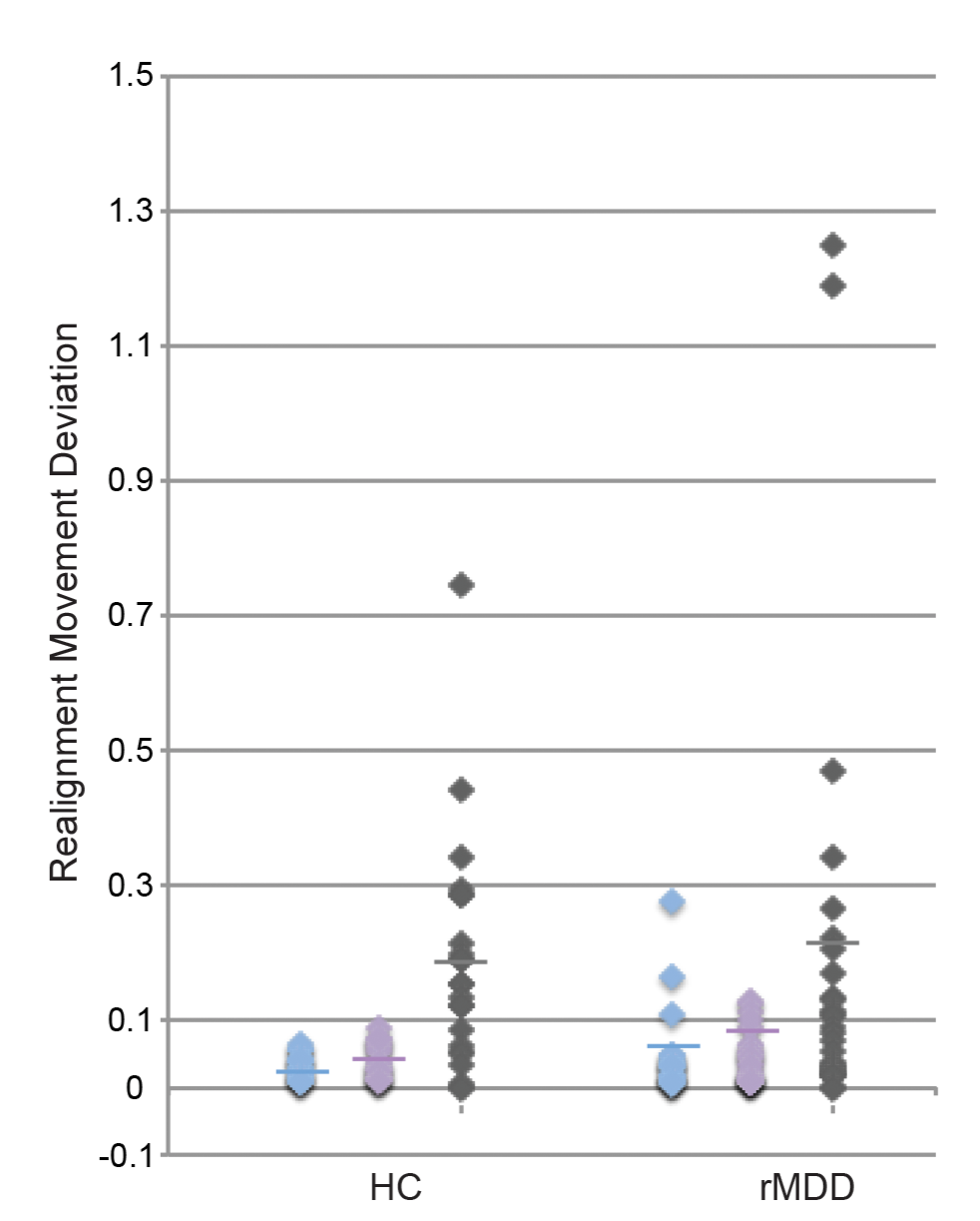

Supplement: Figure S1 — Participants with movement are easily identifiable by movement deviation. Mean movement deviations among the Healthy Control and remitted Major Depressive group in the x, y, and z planes. (TIF) [file pone.0104366.s001.tif]

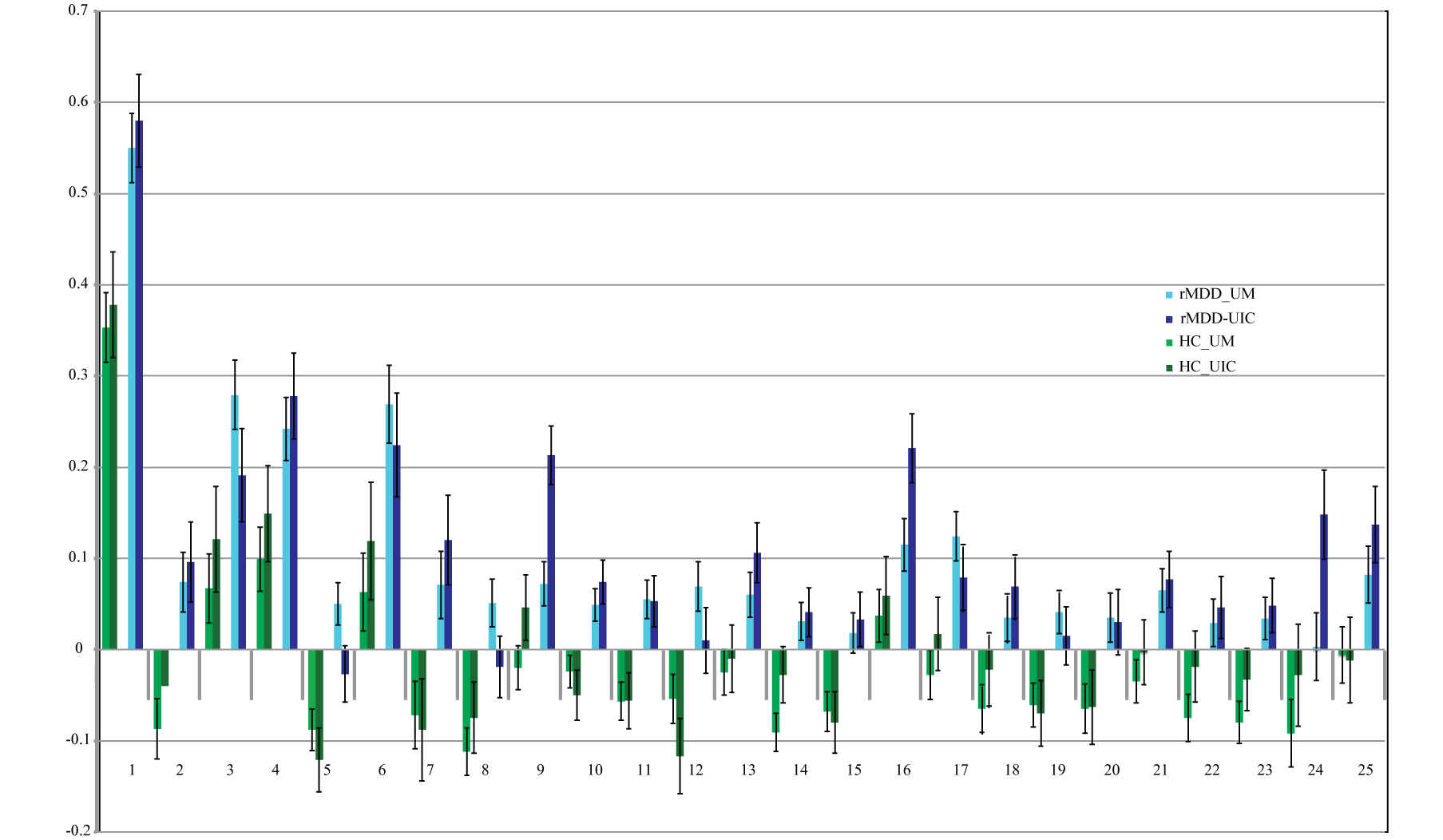

Supplement: Figure S2 — Extracted connectivity bar graphs by cluster, site, and group. rMDD = remitted Major Depressive Disorder; HC = Healthy Control; UM = University of Michigan; UIC = University of Illinois by Chicago. Tailarach coordinates corresponding to each numbered cluster: Clusters 1–7 are connectivities with the left PCC seed, Clusters 8–24 are connectivities with the left sgACC seed, Clusters 25–28 are connectivities with the left amygdala: 1 = −41, 35, 12; 2 = −6, −63, 49; 3 = 12, 45, 14; 4 = 17, −9, 3; 5 = 26, 32, 42; 6 = 27, 24, 20; 7 = 43, −36, 30; 8 = −22, −38, 0; 9 = −24, −30, 16; 9 = −24, 43, 13; 10 = −24, 43, 13; 11 = −31, −83, −33; 12 = −36, 16, 45; 13 = −43, −65, 35; 14 = −54, −54, −1; 15 = −57, −15, −19; 16 = −1, −15, 6; 17 = 8, 32, 49; 18 = 10, 63, 19; 19 = 15, −69, −39; 20 = 29, −71, −39; 21 = 31, −36, 49; 22 = 36, 24, 41; 23 = 43, 14, −3; 24 = 4, 45, 39; 25 = −18, −9, −27; 26 = 19, 12, 21; 27 = 19, 24, 19; 28 = 48, −19, 39. (TIF) [file pone.0104366.s002.tif]
