# Supplementary material for: Increased Coupling of Intrinsic Networks in Remitted Depressed Youth Predicts Rumination and Cognitive Control
Source: PLoS One. 2014 Aug 27;9(8):e104366. doi: 10.1371/journal.pone.0104366 (PMC4146466; doi:10.1371/journal.pone.0104366)
Supplement: File S1 — Supplementary material containing supporting tables. (DOCX) [file pone.0104366.s003.docx]

Supplementary Data

Tables S1 and S2 document significant connectivity differences between rMDD and HC youth using the right PCC and sgACC seeds. Significant connectivity differences overlap with left seeds.

We examined whether there were clinical or demographic differences by site and found that participants at UM had higher VIQ scores (M = 111.21, SD = 8.40) than participants at UIC (M = 105.94, SD = 8.71; *t* = 2.12, *p* = .04). No other clinical or demographic differences were significant across sites.

To rule out several potential confounding factors, we conducted post-hoc exploratory analyses of clinical and technological confounds and examined the influence of participant sex.

*Potential clinical sources of variance and other confounds*

We examined the following clinical sources of variance (residual state effects): residual symptoms on the Ham-D, symptoms on the Ham-A, chronicity assessed as longest MDE duration in weeks, number of years well, age of MDD first-onset, and history of comorbid anxiety disorders. As the current sample is a homogeneous rMDD group by design, restriction of range occurs with many of these variables.

First we examined non-parametric correlations of the clinical sources of variance with hyperconnected clusters within the rMDD group. Longest episode duration was significantly correlated with hyperconnectivity between the left sgACC and the medial frontal gyrus (r = .51, *p* < .05). All other correlations were non-significant. Five participants in the rMDD group had an increase in Ham-D score between phone screen and completion of the clinical interview. We evaluated the significance of group differences after excluding these individuals and results did not change. Within the rMDD group, Ham-D score was correlated with connectivity between the left PCC and the superior middle frontal region (r = 0.44, *p* = .03), whereas the Ham-A was correlated with connectivity between the left sgACC and thalamus (r = 0.42, *p* = .04) and cerebellum (r = -.45, *p* = .03). All significant group differences in connectivity values remained when Ham-D scores were included as a covariate. Last, to test for the effect of a history of comorbid anxiety, we evaluated three diagnostic groups: HC, rMDD, rMDD + Anxiety. All findings in the PCC and sgACC remained, but those in the amygdala were no longer significant. Similarly, when removing all individuals with comorbid anxiety (n = 10), all PCC and sgACC connectivities remained significant, whereas those in the amygdala became non-significant.

*Participant sex as a potential confound*

Our results did not change when evaluating the main effect of sex or when including sex as a covariate.

*Potential technical confounds*

The influence of movement on current findings was evaluated as a potential technical confound in several ways. Two individuals, both in the rMDD group, were identified based on the criterion of greater than .5mm translation of movement in the scan. Excluding these two individuals did not change results using PCC and sgACC seeds, but eliminated significant differences between groups using the amygdala seed. In addition, movement translation values estimated from FSL during realignment were used to evaluate any group differences in movement (including the two outliers). The standard deviations of movement in the x, y, and z planes did not differ between the rMDD and HC group (Table S3, all *p* > .05). A scatterplot of the movement variance in x, y, and z planes is displayed in Figure S1 to illustrate how the individuals with translation greater than .5mm are easily identifiable using movement variance plots.

Scanning site was also evaluated as a potential technical confound. The GE scanner at UM was a Signa scanner and the UIC scanner was a Discovery. The acquisition of forward spiral at UM differed from EPI at UIC. Even though resting state fMRI is thought to be relatively impervious to local scanner specifics, between-site differences could potentially bias any results observed. Figure S2 illustrates extracted connectivity values by site and diagnostic group and suggests that the influence of site on findings is minimal. Of the seed to cluster connectivities that were different between rMDD and HC, none were unduly influenced by site. In all cases, the differences were observed at both sites. Some regions with between group hyperconnectivities to the sgACC were more exaggerated at the UIC site, which may be in part due to greater susceptibility artifact in the orbital frontal cortex with EPI. A general linear model evaluating the effects of site, diagnosis, and the site-by-diagnosis interaction on extracted connectivities indicated an effect of diagnosis (F = 4.42, *p* < .01), but the effect of site (F = 1.7, *p* = .13) and the site-by-diagnosis interaction (F = .88, *p* = .63) were not significant. The only significant site-by-diagnosis post-hoc contrast was for contrast number 25 between the left amygdala and the postcentral gyrus (t = 4.79, *p* = .03).

Table S1. Differences between Healthy Controls and remitted Major Depression for right posterior cingulate seed

| Contrast/lobe |  | BA | Talairach coordinates  x y | | z | Z | mm^3^ |
| --- | --- | --- | --- | --- | --- | --- | --- |
|  | |  |  |  |  |  |  |
|  | |  |  |  |  |  |  |
| Frontal  Middle frontal    Medial frontal  Precentral    Inferior frontal  Limbic | | 8  8  4  9  47 | -29  -4  20  48  -41 | 16  41  -19  1  28 | 36  39  52  19  -9 | 3.04  4.17  3.54  3.93  3.53 | 1296  8488  1392  448  752 |
| Cingulate  Posterior cingulate    Temporal  Fusiform  Inferior temporal | | 31  30  20  20 | -6  -29  -48  34 | -52  -63  -25  -7 | 29  12  -24  -38 | 5.85  3.81  4.04  3.65 | 5848  4648  6112  616 |
| Subcortical  Insula  Parietal | |  | -27 | 16 | 20 | 3.8 | 1176 |
| Inferior parietal | | 40 | -32 | -46 | 45 | 3.52 | 584 |

*Note.* BA = Brodmann’s Area; mm = millimeter

Table S2. Differences between Healthy Controls and remitted Major Depression for right subgenual anterior cingulate

| Contrast/lobe |  | BA | Talairach coordinates  x y | | z | Z | mm^3^ |
| --- | --- | --- | --- | --- | --- | --- | --- |
|  | |  |  |  |  |  |  |
|  | |  |  |  |  |  |  |
| Frontal | |  |  |  |  |  |  |
| Inferior frontal    Limbic  Parahippocampus | | 46  36 | -29  27 | 41  -23 | 9  -26 | 3.27  3.61 | 968  464 |
| Temporal  Middle temporal | | 21 | 57 | -56 | 2 | 3.25 | 512 |
|  | |  |  |  |  |  |  |
| Subcortical  Thalamus  Parietal  Inferior parietal    Posterior  Pyramis  Inferior semi-lunar lobule  Declive | | 7 | -8  -36  -13  10  -54 | -23  -52  -87  -79  -63 | 11  45  -33  -34  -19 | 4.51  3.07  4.01  3.61  3.31 | 2744  504  3072  1512  448 |

*Note.* BA = Brodmann’s Area; mm = millimeter

Table S3. Movement deviations in the x, y, and z planes do not differ between rMDD and HCs

| \| Plane \| Diagnosis \| Mean SD \| SD \| \| --- \| --- \| --- \| --- \| \| mm X \| HC \| 0.03 \| 0.02 \| \|  \| rMDD \| 0.04 \| 0.05 \| \| mm Y \| HC \| 0.04 \| 0.02 \| \|  \| rMDD \| 0.04 \| 0.04 \| \| mm Z \| HC \| 0.21 \| 0.17 \| \|  \| rMDD \| 0.20 \| 0.30 \| |
| --- | --- | --- | --- | --- | --- | --- | --- | --- | --- | --- | --- | --- | --- | --- | --- | --- | --- | --- | --- | --- | --- | --- | --- | --- | --- | --- | --- | --- |

*Note*. mm = millimeter; rMDD = remitted Major Depressive Disorder; HCs = Healthy Controls; SD = Standard Deviation
